# Supplementary material for: CD8+ T Cell Phenotype and Function in Childhood and Adult-Onset Connective Tissue Disease
Source: Int J Mol Sci. 2022 Sep 28;23(19):11431. doi: 10.3390/ijms231911431 (PMC9569696; doi:10.3390/ijms231911431)
Supplement: Supplementary file 1 [file ijms-23-11431-s001.zip › ijms-1884867-supplementary.pdf]

Table S1. Altered peripheral blood $CD8^+$ T cell phenotype in adults with SLE.

Author et al., year [ref]	Type of study	N: patients or controls<br>Age[1]: Mean ± SD or median (range) or [IQR]	$CD8^+$ T cell populations	Clinical relevance
Wiechmann A. et al., 2021 [72]	Cross-sectional	N= 31 SLE<br>42.5 ± 13.7<br>N=21 HC<br>38.2 ± 14.4	Decreased % $CD8^+CD107a^+$ T cells in SLE vs HC ($p = 0.02$).	Decreased % $CD8^+CD107a^+$ T cells associated with absence of LN ($p = 0.01$), inactive disease ($p = 0.003$) and HCQ treatment in combination with either prednisolone ($p = 0.02$) or prednisolone and other DMARDs ($p = 0.004$). Positive correlation between % $CD8^+CD107a^+$ T cells and SLEDAI (r = 0.5, $p < 0.005$). Negative correlation between % $CD8^+CD107a^+$ and HCQ dose (r = –0.5, $p = 0.005$).
Lima G. et al., 2021 [49]	Cross-sectional	N=30 SLE<br>15 active + 15 inactive<br>33 (30–46) active SLE<br>49 (48–60) inactive SLE<br>N=29 HC<br>43 (32–52)	No difference in % $CD3^+CD8^+$ T cells in SLE (regardless of disease activity) vs HC.<br>No difference in % $CD8^+$ TEMRA population between HC and SLE.<br>Increased cell activation ($CD38^+$HLA-DR^+, $p < 0.01$) and exhaustion ($CD38^+$HLA-DR^+PD-1^+, $p < 0.001$) T cell markers in SLE vs HC.	Reduced % naïve $CD8^+$ T cells in inactive SLE vs active SLE ($p < 0.05$).<br>Increased % CM $CD8^+$ T cells in inactive SLE vs HC and active SLE ($p < 0.01$).<br>Reduced % EM $CD8^+$ T cells in active SLE vs HC and inactive SLE ($p < 0.05$).
Lu Z. et al., 2019 [53]	Cross-sectional	N=39 SLE<br>36.4 ± 13.5<br>N=25 HC<br>ASM	Increased % of $CD8^+$ T cells in SLE compared to HC ($p = 0.029$). Decreased absolute $CD8^+$ T cell counts ($p < 0.01$) and CD4/CD8 T cell ratios ($p = 0.001$) in SLE vs HC.	All patients were treatment naïve.
Minning S. et al. 2019 [69]	Cross-sectional with 28-day follow-up for limited	N=47 SLE<br>Mean Age: 32.7 (19–68)<br>N=20 HC<br>Mean age: 37	Decreased % $CD8^+CD28^+$ T cells and $CD8^+CD28^-/CD8^+CD28^+$ T cell ratio in high-activity SLE vs HC and inactive SLE ($p < 0.05$).<br>Increased % $CD8^+CD28^-$ T cells in inactive SLE patients vs HC ($p < 0.05$).	Inverse correlation between % $CD8^+CD28^+$ T cells and SLEDAI (r = –0.5, p < 0.01). Positive correlation between $CD8^+CD28^-/CD8^+CD28^+$ ratio and SLEDAI (r = 0.45, p < 0.01). Decreased % $CD8^+CD28^+$ in SLE patients with a reduced C3 level ($p < 0.05$). $CD8^+CD28^-/CD8^+CD28^+$

	subset of patients			ratio higher in patients with a reduced vs normal C3 levels and higher in patients with vs without kidney damage ($p < 0.05$). % $CD8^+CD28^+$ and $CD8^+CD28^-/CD8^+CD28^+$ ratio normalised 28 days post high dose steroids ± immunosuppressants.
Gao H. et al., 2018 [59]	Cross-sectional	N= 40 SLE<br>29.41 ± 6.28<br>N= 40 HC<br>29.70 ± 5.99	Increased % $CD8^+$ T cells in SLE vs HC (p < 0.001).	Positive correlation between % $CD8^+$ T cells and RF level (r = 0.50, $p = 0.002$). Negative correlation between % $CD8^+$ cells and C3 (r = −0.374, $p = 0.017$) and C4 (r = −0.509, $p = 0.001$).
Manjarres-Orduno N. et al., 2017 [63]	Cross-sectional	N=44 SLE<br>38.5 ± 10.41<br>N=40 HC<br>39.3 ± 9.42	No difference in naïve ($CD45RA^+CCR7^+$), CM ($CD45RA^-$, $CCR7^+$) or early memory ($CCR7^-CD28^+$) subsets, between HC and SLE. Increased % TEMRA ($CCR7^-CD45RA^+CD28^-$) cells in SLE vs HC ($p = 0.02$). Patients with increased % of these cells had a "cytotoxic phenotype" (higher production of IFN-γ, perforin, & granzyme B) across all stages of T cell differentiation.	Increased % of terminally differentiated $CD8^+$ T cells associated with LN ($p < 0.02$).
Mortezagholi S. et al., 2016 [54]	Cross-sectional	N=35 SLE<br>37.17±11.04<br>N=38 HC<br>ASM	Increased % of $CD8^+$ T cells in SLE vs HC ($p < 0.001$).<br>Decreased CD4/CD8 ratio in SLE vs HC ($p = 0.009$).<br>Increased TLR9 expression on $CD8^+$ T cells in SLE vs HC ($p = 0.001$).	None reported.
Kim J-S. et al., 2012 [71]	Cross-sectional	N=21 SLE<br>35.8 ± 9.3<br>N=23 HC<br>33.3 ± 7.0	Increased % of cytotoxic IL7Rα^{low} cells in SLE ($p = 0.027$).	Positive correlation between % of IL7Rα^{low} EM $CD8^+$ cells and SLEDAI score (r = 0.483, $p = 0.026$).
Suarez-Fueyo A. et al., 2011 [55]	Cross-sectional	N=55 SLE<br>Age range: 25–58<br>N=31 HC<br>Age range: 24–55	Increased % $CD8^+$ T cells in SLE vs HC ($p < 0.01$). Increased % memory $CD8^+CD45RO^+$ cells in inactive ($p < 0.01$) and active SLE ($p < 0.001$) vs HC.	None reported.

Shah D. et al., 2011 [70]	Cross-sectional	N=26 SLE<br>28.5 ± 7.50<br>N=26 HC<br>26.90 ± 8.0	Increased % $CD8^+$ granzyme B^+ T cells ($p < 0.001$), % $CD8^+$ $perforin^+$ T cells ($p < 0.001$) and % $CD8^+$ T cells co-expressing granzyme B and perforin ($p < 0.001$) in SLE vs HC. Increased serum granzyme B in SLE vs HC ($p < 0.001$).	Positive correlation between SLEDAI score and % $perforin^+$ $CD8^+$ ($r = 0.543$, $p < 0.01$), % granzyme B^+ $CD8^+$ ($r = 0.508$, $p < 0.01$), % $perforin^+$ granzyme B^+ $CD8^+$ cells ($r = 0.705$, $p < 0.001$). Negative correlation between serum granzyme B and C3 ($r = -0.510$, $p < 0.01$) and C4 ($r = -0.583$, $p < 0.01$). Positive correlation between serum granzyme B and SLEDAI ($r = 0.783$, $p < 0.001$).
Shah D. et al., 2011 [58]	Cross-sectional	N=35 SLE<br>28.5 ± 7.80<br>N=35 HC<br>28.25 ± 6.80	Increased % $CD8^+$ T cells in SLE vs HC ($p < 0.001$). Decreased $CD4^+$ /$CD8^+$ T cell ratio in SLE vs HC ($p < 0.001$).	None reported.
Dolff S. et al., 2010 [62]	Cross-sectional	N=43 SLE<br>41±12<br>N=20 HC<br>36±10	Increased % $CD8^+$ CM ($CCR7^+CD45RO^+$) in SLE vs HC ($p = 0.02$).<br>Decreased % $CD8^+$ EM in SLE vs HC ($p = 0.008$).<br>No difference in % naïve $CD8^+$ T cells.	Increased % $CD8^+$ CM in active disease vs HC ($p = 0.009$) and SLE without active LN ($p = 0.02$).<br>Decreased % CD8 EM in active SLE vs HC ($p = 0.004$), reduced in those without active LN ($p = 0.03$) and lowest in those with active LN ($p = 0.01$).
McKinney E.F. et al., 2010 [64]	Longitudinal prospective	N=29 SLE (7 poor prognosis, 18 better prognosis)<br>Mean age (SEM) poor prognosis: 42.6 (4.3)<br>Mean age (SEM) better prognosis: 43.3 (3.2)	Increased % $CD3^+CD8^+CD45RA^-$ ($p = 0.039$) and absolute count ($p = 0.003$) of memory cells (both EM and CM) in SLE with poor prognosis.	Increased % and number of $CD8^+$ memory cells is associated with lower flare-free survival time in SLE.
Bohm I. 2006 [60]	Cross-sectional	N= 124 LE (SLE+ cutaneous LE)<br>Mean age: 43.7 (8–84)<br>N=57 HC	Decreased absolute $CD3^+CD8^+$ T cell count in LE compared to HC ($p < 4 \times 10^{-11}$).	None reported.

		Mean Age: 40.8 (11–84)		
Wang H. et al., 2005 [51]	Cross-sectional	N=49 SLE<br>Mean age: 25.3<br>N=30 HC<br>AM	No differences in % of CD8⁺ T cells SLE vs HC. Decreased CD4/CD8 T cell ratio in SLE (both active and inactive) vs HC ($p < 0.05$).	No difference in % $CD8^+$ cells between active and inactive SLE.
Wouters C.H.P. et al., 2004 [52]	Cross-sectional	N=25 SLE<br>34.5 (17.4–56.5)<br>N=38 HC	No difference in % $CD8^+$ T cells between SLE vs HC.<br>Decreased absolute $CD8^+$ T cell counts in SLE vs HC ($p < 0.01$).<br>Increased % of activated $CD8^+$ T cells ($CD8^+HLA\text{-}DR^+$) in SLE vs HC ($p < 0.05$).	SLE patients were not on corticosteroids or immunosuppressive therapy. Distribution of lymphocyte subsets remained the same between HCQ treated and untreated groups.
Sen Y. et al., 2004 [124]	Cross-sectional	N= 37 SLE<br>31.4 (21–48)<br>N= 18 HC<br>30.5 (18–48)	Increased % $CD8^+CCR7^+$ T cells in active SLE vs inactive SLE and HC ($p < 0.01$). These are mostly CM cells ($CD45RO^+$ expression over 90% in all groups).	Increased % of CM $CD8^+$ cells seen in active lupus only.
Viallard J.F. et al., 2001 [67]	Longitudinal prospective	N=34 SLE with quiescent disease,<br>Mean age: 37.9 (16–74)<br>N=26 SLE with active disease<br>Mean Age: 38.0 (16–79)	Increased % $CD8^+HLA\text{-}DR^+$ T cells in active vs inactive SLE ($p = 0.0000001$). No difference in absolute counts.	No differences in % $CD8^+HLA\text{-}DR^+$ T cells between group receiving treatment and untreated patients. In multivariate analysis, % $CD8^+HLA\text{-}DR^+$ was found to be the best biological parameter associated with flaring ($p < 0.001$).
Hu S. et al., 2001 [57]	Cross-sectional	N=21 active SLE<br>29.14 ±7.82<br>N=10 HC<br>32.07 ± 8.73	Increased % of $CD8^+$ T cells in SLE vs HC ($p < 0.01$). Decreased CD4/CD8 T cell ratio in SLE vs HC ($p < 0.01$).	None reported.

[1]Age in years expressed in this format, unless specified otherwise.

Abbreviations used: AM = age matched, ASM = age and sex matched, CM = central memory, C3 = complement 3, C4 = complement 4, DMARDs = disease-modifying antirheumatic drugs, EM = effector memory, HC = healthy controls, HLA-DR = Human Leukocyte Antigen-DR, HCQ = hydroxychloroquine, IFN = interferon, IL7R = interleukin-7-receptor IQR = interquartile range, LE = lupus erythematosus, LN = lupus nephritis, PD-1 = programmed cell death protein 1, RF = rheumatoid factor, SD =

standard deviation, SEM = standard error of the mean, SLE = systemic lupus erythematosus, SLEDAI = Systemic Lupus Erythematosus Disease Activity Index, TEMRA = Terminally differentiated effector memory cells, TLR = Toll-like receptor.

Table S2. Altered peripheral blood CD8+ T cell phenotype in adults with connective tissue disease.

**Author et al., year [ref]**	**Type of study**	**N: patients or controls Age[1]: Mean ± SD or median (range) or [IQR]**	**Altered CD8+ T cell phenotype**	**Clinical relevance**
**Sjogren's syndrome (SS)**				
Wu C. et al., 2018 [80]	Cross-sectional	N=32 pSS<br>54 (43.75–64)<br>N=23 SLE<br>34 (18–74)<br>N= 20 HC<br>42 (33.75–46.25)	Increased % $CCR7^+CD8^+$ cells in pSS and SLE vs HC ($p < 0.05$) Higher chemotactic motility of $CD8^+$ cells T cells in pSS and SLE vs HC ($p < 0.05$).	None reported.
Sudzius G. et al., (2015) [75]	Cross-sectional	N=52 pSS (n=29 pSS Ab−ve, n=23 pSS Ab+ve)<br>57 ± 13<br>N=28 HC<br>53 ± 11	No difference in % $CD8^+$ T cells in pSS vs HC. Decreased absolute $CD8^+$ T cell count in pSS vs HC ($p < 0.01$). Decreased absolute count of $CD8^+CD57^-CD27^+$ T cells ($p < 0.01$) in pSS vs HC.	Positive correlation between focus score and absolute counts of $CD8^+CD57^-CD27^+$ T cells (r = 0.560, $p = 0.016$) in SS Ab−ve patients.<br>In SS Ab+ve patients: Positive correlation between Schirmer's I test and % $CD8^+CD57^-CD27^+$ T cells (r = 0.589, $p = 0.006$) and negative correlation with % $CD8^+CD57^-CD27^-$ T cells (r = −0.479, $p = 0.033$). Positive correlation between Schirmer's I test and $CD8^+CD57^-CD27^+$ absolute T cell count (r = 0.512, $p = 0.021$) and negative correlation with $CD8^+CD57^-CD27^-$ T cell count (r = −0.525, $p = 0.017$). Positive correlation between unstimulated salivary flow rate and % $CD8^+$ T cell (r = 0.525, $p = 0.017$). Negative correlation between focus score and % and absolute counts of $CD8^+CD57^+CD27^-$ T cells, respectively, (r = −0.517, $p = 0.02$) and (r = −0.462, $p = 0.04$). Negative correlation between ESSDAI and absolute counts of $CD8^+$ T cells (r = −0.459, $p = 0.042$) and $CD8^+CD57^+CD27^+$ (r = −0.492, $p = 0.028$) T cells.

Fauchais A. et al., 2009 [77]	Cross-sectional	N= 18 SS<br>57 (21–83)<br>N= 12 HC, ASM	Increased activated HLA-DR$^+$CD8$^+$ T cell absolute cell count in pSS vs HC (p = 0.02).	None reported.
**Systemic scleroderma (SSc)**				
Gambichler T. et al., 2010 [84]	Cross-sectional	N=29 SSc (22 lcSSc, 7 dcSSc)<br>56.3 ± 12.9<br>N=29 HC<br>51.1 ± 13.4	No difference in % CD8$^+$ T cells in SSc vs HC. Decreased absolute CD8$^+$ T cell count in SSc vs HC ($p = 0.021$).	None reported.
Hussein M. et al., 2005 [83]	Cross-sectional	N= 19 SSc<br>34.8±2.6<br>N=6 HC<br>33.03 ± 7.2	No difference in CD8$^+$ cell count in SSc vs HC. Increased CD4$^+$/CD8$^+$ ratio in SSc vs HC ($p < 0.01$).	None reported.
Tiev K. et al., 2004 [82]	Cross-sectional	N= 11 dcSSc<br>Mean age: 48<br>SEM ±4<br>N=11 lcSSc<br>Mean age: 54<br>SEM ±2<br>N=11 HC<br>Mean age: 52<br>SEM±2	No difference in % activated HLA-DR$^+$CD8$^+$ or CD25$^+$CD8$^+$ T cells in SSc vs HC. Decreased % CD8$^+$ in dcSSc vs HC ($P < 0.05$). No difference in % CD8$^+$ T cells in lcSSc vs HC. No significant CD8$^+$ T cell repertoire perturbations in SSc vs HC.	None reported.
Tsuji-Yamada J. et al., 2001 [81]	Cross-sectional	N = 23 SSc (12 lcSSc, 11 dcSSc)<br>58 ± 8.9<br>N= 14 HC<br>52.2 ± 3.6	Increased % CD8$^+$ T cells in SSc vs HC ($p < 0.05$). Increased % CD8$^+$IL-4$^+$ T cells ($p < 0.05$) in SSc vs HC. Decreased % CD8$^+$IL-2$^+$ T cells in SSc vs HC ($p < 0.001$).	No difference in % CD8$^+$ T cells with age, treatment, ANA type, extent of skin sclerosis, presence of pulmonary fibrosis, or oesophageal dysfunction. Negative correlation between IL-4/IL-4 $^+$IL-2 producing CD8$^+$ T cells and SSc duration ($r = –0.526$, $p = 0.01$).
**Polymyositis and Dermatomyositis (PM/DM)**				
Wilkinson M. et al., 2020 [90]	Cross-sectional	N=19 DM<br>55.8 [46.21–60.3]<br>N=9 PM<br>52.37 [48.35–53.10]	Decreased % CD8$^+$ CM T cells in PM vs HC ($p = 0.0093$). No difference in %CD8$^+$ T cells in DM/PM vs HC.	No association with treatment or disease activity. Decreased % CD8$^+$ CM in patients with anti-Ro, anti-PM/Scl and anti-RNP antibodies.

		N=25 HC<br>51.26<br>[43.23–58.31]		
Benveniste O. et al., 2001 [91]	Cross-sectional	N= 10 PM<br>58.8 ± 16.3<br>N= 10 DM<br>49.7 ± 20.4<br>N=17 HC<br>32±9	Perturbations in $CD8^+$ T cell repertoire in PM vs DM and PM vs HC due to oligoclonal $CD8^+$ T cell expansion.	None reported.

[1]Age in years expressed in this format, unless specified otherwise.

Abbreviations used: Ab = antibody, ANA = antinuclear antibody, ASM = age and sex matched, CM = central memory, CCR = CC-chemokine receptor, DM = dermatomyositis, dcSSc = diffuse cutaneous systemic sclerosis, ESSDAI = EULAR Sjögren's syndrome disease activity index, , HC = healthy controls, HLA-DR = Human Leukocyte Antigen-DR, IQR = interquartile range, IL= Interleukin, , lcSSc = limited cutaneous systemic sclerosis, PM = polymyositis, pSS = primary Sjögren's syndrome, RN*P* = ribonucleoprotein, SD = standard deviation, SEM = standard error of the mean, SLE = systemic lupus erythematosus, SS = Sjögren's syndrome, SSc = systemic sclerosis.
